# Rethinking Equine Cystitis: Evidence from Four Atypical Clinical Presentations

**DOI:** 10.3390/life16081285

**Published:** 2026-08-04

**Authors:** Anna Biazik, Magdalena Sobuś, Radomir Henklewski, Michał Wieteska, Paweł Kordowitzki

**Affiliations:** 1 Department of Veterinary Surgery, Institute of Veterinary Medicine, Faculty of Biological and Veterinary Sciences, Nicolaus Copernicus University in Torun, ul.Gagarina 7, 87-100 Torun, Poland; 2 Department of Diagnostics and Clinical Sciences, Institute of Veterinary Medicine, Faculty of Biological and Veterinary Sciences, Nicolaus Copernicus University in Torun, ul.Gagarina 7, 87-100 Torun, Poland; 3 Department of Basic and Preclinical Sciences, Institute of Veterinary Medicine, Faculty of Biological and Veterinary Sciences, Nicolaus Copernicus University in Torun, ul.Gagarina 7, 87-100 Torun, Poland

**Keywords:** bladder, sabulous cystitis, Equus caballus, inflammation, *Facklamia* spp.

## Abstract

In this case series, four equines were diagnosed with cystitis, resulting in divergent clinical outcomes. Three animals achieved complete clinical resolution without recurrence, whereas one required euthanasia due to progressive clinical deterioration. All diagnoses were established through a dual-modality investigative protocol comprising cystoscopic evaluation and histopathological examination of bladder mucosal biopsies. The cohort exhibited considerable etiological heterogeneity. One case was classified as chronic idiopathic cystitis, while another was attributed to an opportunistic bacterial infection involving *Facklamia* spp. A third case demonstrated histopathological features consistent with mild chronic-active inflammation, characterized by mucosal hyperplasia and focal ulceration. Of particular pathological significance was one equine presented with marked epithelial denudation accompanied by pronounced submucosal edema, notably in the absence of appreciable inflammatory cellular infiltration. These characteristic morphological features and lesions observed in the horses share certain histopathological features with inflammatory bladder lesions described as interstitial cystitis in other species. However, these conclusions should be interpreted with caution, as the findings are based on only four horses.

## 1. Introduction

Cystitis in horses is most often secondary to a coexisting urinary outflow problem, such as bladder stones, bladder paralysis, a tumor or anatomical defect, or other factors [1,2,3,4,5,6]. Risk factors include trauma to the urinary tract, frequent catheterization, vaginal infections in mares [1], and a history of parturition [6]. Cystitis in horses can also result from the use of certain medications, such as cyclophosphamide or phenylbutazone [6]. Cystitis has been reported in horses grazing on pastures predominantly composed of sorghum, Sudan grass, or sorghum-Sudan grass hybrids. Ascending infections of the urinary tract and urinary bladder are reported more frequently in mares due to their relatively short urethra [4,7]. Primary cystitis is rare in horses, unlike in other species [8,9,10]. Feline Urologic Syndrome (FUS), now generally referred to as Feline Lower Urinary Tract Disease (FLUTD), is one of the most frequently diagnosed urinary tract disorders in cats [11].

It is a collection of conditions affecting a cat’s bladder and urethra. The most common clinical signs are difficulty or pain during urination, frequent urination, and sometimes urinating outside the litter box. While the exact causes are often unknown, potential factors include bladder stones, urinary tract infections, and stress. The most common pathogens isolated from patients with cystitis are Gram-negative: *Escherichia coli*, *Pseudomonas aeruginosa*, *Klebsiella* spp., *Proteus* spp., and Gram-positive: *Staphylococcus* spp. and *Streptococcus* spp. [12]. There are only a few cases of cystitis caused by less common pathogens such as *Facklamia* sp.

Interstitial cystitis (IC) is another possible differential diagnosis when the described signs occur in both humans and animals. It is estimated that up to one million people in the United States have interstitial cystitis, also called painful bladder syndrome [13]. The symptoms may resemble those of urinary tract infections, but clinical signs are absent, urine cultures reveal no microorganisms, and the disease does not respond to antibiotic treatment [14,15,16,17]. The diagnosis of IC is difficult; urinary tract infections, cancer, bladder inflammation, kidney stones, and other types (granulomatous, eosinophilic) of cystitis must be excluded. Typically, equine cystitis manifests with observable clinical signs such as dysuria (painful urination), pollakiuria (increased frequency of urination), stranguria (straining to urinate), hematuria (blood in urine), and urinary incontinence, particularly evident in conditions like sabulous cystitis, where the accumulation of calcium carbonate crystals irritates the bladder mucosa [2,11]. Diagnostic approaches often involve a combination of clinical examination and urinalysis to detect abnormalities such as alkaline pH, pyuria, and hematuria, as well as imaging techniques such as ultrasonography and cystoscopy to visualize bladder lesions or uroliths [2,12,13].

The aim of this study was to retrospectively review several interesting and atypical cases of cystitis in horses. Histopathological findings were inconclusive and frequently resembled lesions more commonly described in other species rather than in horses. This study highlights the diagnostic challenges associated with cystitis in horses, including the diversity of clinical presentations, the predominance of nonspecific clinical signs, and the need for a comprehensive diagnostic work-up. It also emphasizes the importance of a therapeutic approach that accounts for concurrent diseases to achieve an appropriate and successful clinical outcome. With the presentation of the four case reports, we further aim to stimulate new research projects to shed more light on the interesting topic of urinary tract diseases in horses.

## 2. Materials and Methods

### 2.1. Case Selection

This retrospective case series describes four horses diagnosed with cystitis and examined at a specialized equine referral practice in Poland. Cases were included when cystoscopic examination identified macroscopic abnormalities of the urinary bladder mucosa and histopathological evaluation of endoscopically obtained bladder biopsies confirmed inflammatory or degenerative changes. Clinical records, endoscopic findings, laboratory investigations, histopathological reports, treatments administered, and follow-up information were reviewed and analyzed.

### 2.2. Clinical Examination and Diagnostic Work-Up

All horses underwent a comprehensive physical examination upon admission, including assessment of heart rate, respiratory rate, rectal temperature, body condition, and clinical signs associated with urinary tract dysfunction. Particular attention was paid to abnormalities occurring during exercise, urination behavior, urinary incontinence, pollakiuria, lethargy, and performance-related issues. Since several horses presented with clinical signs potentially attributable to Equine Gastric Ulcer Syndrome (EGUS), gastroscopic examination was performed when clinically indicated. Gastric lesions were assessed and graded according to established scoring systems for equine gastric ulceration. The non-glandular region was assessed using the widely accepted grading system described in 1999 [18], in which grade 1 corresponds to an intact mucosal surface. Lesions of the glandular region were evaluated using the descriptive scoring system based on the consensus statement [19].

### 2.3. Sedation Protocol and Endoscopic Procedures

Cystoscopy and gastroscopy were performed under standing sedation. Depending on the individual case, horses received acepromazine (0.05 mg/kg intravenously) (Tranquinervin^®^ 10 mg/mL, Produlab Pharma B.V., Raamsdonksveer, The Netherlands), detomidine (0.012 mg/kg intravenously) (Domidine^®^ 10 mg/mL, Eurovet Animal Health B.V., Handelsweg, The Netherlands), and butorphanol (0.012 mg/kg intravenously) (Torbugesic^®^ 10 mg/mL, Zoetis Manufacturing, Madrid, Spain). In some cases, acepromazine was omitted at the clinician’s discretion. A Karl Storz flexible videoendoscope (length 140 cm, sheath diameter 9.7 mm) was used to visualize the urinary bladder. The entire bladder cavity was systematically examined, including the bladder neck, corpus vesicae, and apex vesicae. Particular attention was paid to the presence of mucosal abnormalities, ulceration, hyperemia, hemorrhage, raised lesions, epithelial defects, and accumulation of urinary sediment or sand. Representative biopsy specimens were collected from abnormal mucosal regions using endoscopic biopsy forceps. In cases requiring follow-up evaluation, repeat cystoscopy and additional biopsy sampling were performed to assess lesion progression and response to therapy.

### 2.4. Histopathological Examination

Biopsy specimens obtained during cystoscopy were fixed in 10% neutral-buffered formalin, routinely processed, embedded in paraffin, sectioned, and stained with hematoxylin and eosin (H&E). The cystoscopic biopsy specimens were small and superficial and therefore did not permit evaluation of the full thickness of the bladder wall. Histopathological evaluation was performed by a European College-certified veterinary pathologist. The examined tissues were assessed for epithelial integrity, inflammatory infiltrates, ulceration, fibrosis, edema, vascular changes, cellular degeneration, mucosal hyperplasia, and evidence of neoplastic transformation. Particular attention was given to the characterization of inflammatory cell populations, including lymphocytes, plasma cells, macrophages, mast cells, and neutrophils. Histopathological diagnoses were established on the basis of the predominant tissue alterations observed in each specimen. The present observations should be interpreted as descriptions of mucosal and superficial bladder lesions identified in endoscopically obtained biopsy specimens.

### 2.5. Microbiological and Molecular Investigations

In the horse presenting with severe inflammatory lesions and suspected bacterial cystitis, urine samples were submitted for bacteriological culture in an external commercial laboratory. Following isolation of Gram-positive cocci, bacterial identification was performed by sequencing the 16S rRNA gene fragment. Sequence data were compared with entries in the National Center for Biotechnology Information (NCBI) database for species-level identification. For molecular identification of the bacterial isolate, a 16S rRNA gene fragment was analyzed by Sanger sequencing. The resulting chromatogram files were manually inspected, and the nucleotide sequence was corrected as necessary based on the quality of the chromatographic signals. The final sequence analyzed was 776 bp in length. The obtained sequence was compared with sequences deposited in the NCBI Core Nucleotide database using the BLASTN algorithm, with the “Highly similar sequences (megablast)” option selected. Search results were ranked according to E-value. Species-level identification was considered when the analyzed 16S rRNA gene sequence showed ≥99% nucleotide identity to a reference sequence and contained <1% ambiguous nucleotide positions. The selected identity threshold was based on common recommendations concerning the use of 16S rRNA gene sequence identity for prokaryotic taxonomic classification.

### 2.6. Additional Diagnostic Procedures

When chronic inflammatory lesions were identified without a clear infectious etiology, additional investigations were undertaken to identify potential contributing factors. Selected horses underwent food allergy testing performed by independent commercial laboratories using serological methods based on immunoglobulin E (IgE), immunoglobulin G (IgG), PAX, and Next+ methodologies. Although the diagnostic value of allergy testing in horses remains controversial, the results were used as supportive information when formulating individualized dietary recommendations. The results of IgE-, IgG-, PAX-, and related testing were considered supportive information only and were not interpreted as definitive evidence of food allergy.

### 2.7. Therapeutic Management and Follow-Up

Treatment protocols were individualized according to clinical presentation and diagnostic findings. Therapeutic interventions included antiulcer medication omeprazole (Equinor^®^ 370 mg/g, Norbrook Laboratories Limited, Leicestershire, UK), misoprostol (Cytotec^®^ 200 mcg tabl., Pfizer, Pearl River, NY, USA), and sucralfate (Ulgastrol^®^, Hipppovet Pharmacy, Malin, Poland), anti-inflammatory therapy (meloxicam Contacera^®^, Zoetis, Louvain-la-Neuve, Belgium; Dublin, Ireland), antimicrobial treatment (trimethoprim-sulfamerazine Trimerazin^®^, Biowet Drwalew Sp. z o.o., Drwalew, Poland; 400 mg + 80 mg, Biowet Drwales, Poland), bladder lavage with sterile isotonic saline, dietary modification, and administration of a commercially available herbal preparation (Phytolysin^®^, Polpharma S.A., Starogard Gdanski, Poland), a herbal preparation (containing wheatgrass, birch leaf, fenugreek seeds, parsley root, goldenrod, horsetail herb, lovage root, knotgrass herb, and onion flakes) which has anti-inflammatory and diuretic effects and is used in human medicine (Polpharma, Starogard Gdanski, Poland). Dietary interventions included reducing dietary starch and sugar intake, unrestricted access to forage, elimination of potential dietary allergens, modifying mineral intake, and supplementing with wheat bran and vitamin C when clinically indicated.

### 2.8. Ethical Considerations

All diagnostic and therapeutic procedures were performed as part of routine clinical management and were undertaken with informed owner consent. No experimental interventions were conducted, and all procedures complied with accepted standards of veterinary clinical practice in Poland. Ethical approval was not required, as the procedures were considered non-experimental clinical veterinary practices, in accordance with Polish and EU law (Dz. U. 2015 poz. 266 and 2010-63-EU directive). The horses were patients at the Institute of Veterinary Medicine, Nicolaus Copernicus University in Torun, Poland, under the routine medical care and treatment of one of the authors, who diagnosed and monitored the horses in the clinic or in the field. The horses’ owners had been informed in advance, and they consented to the use of the study’s results in the article.

## 3. Case Presentations

### 3.1. Case One

**Figure 1:**
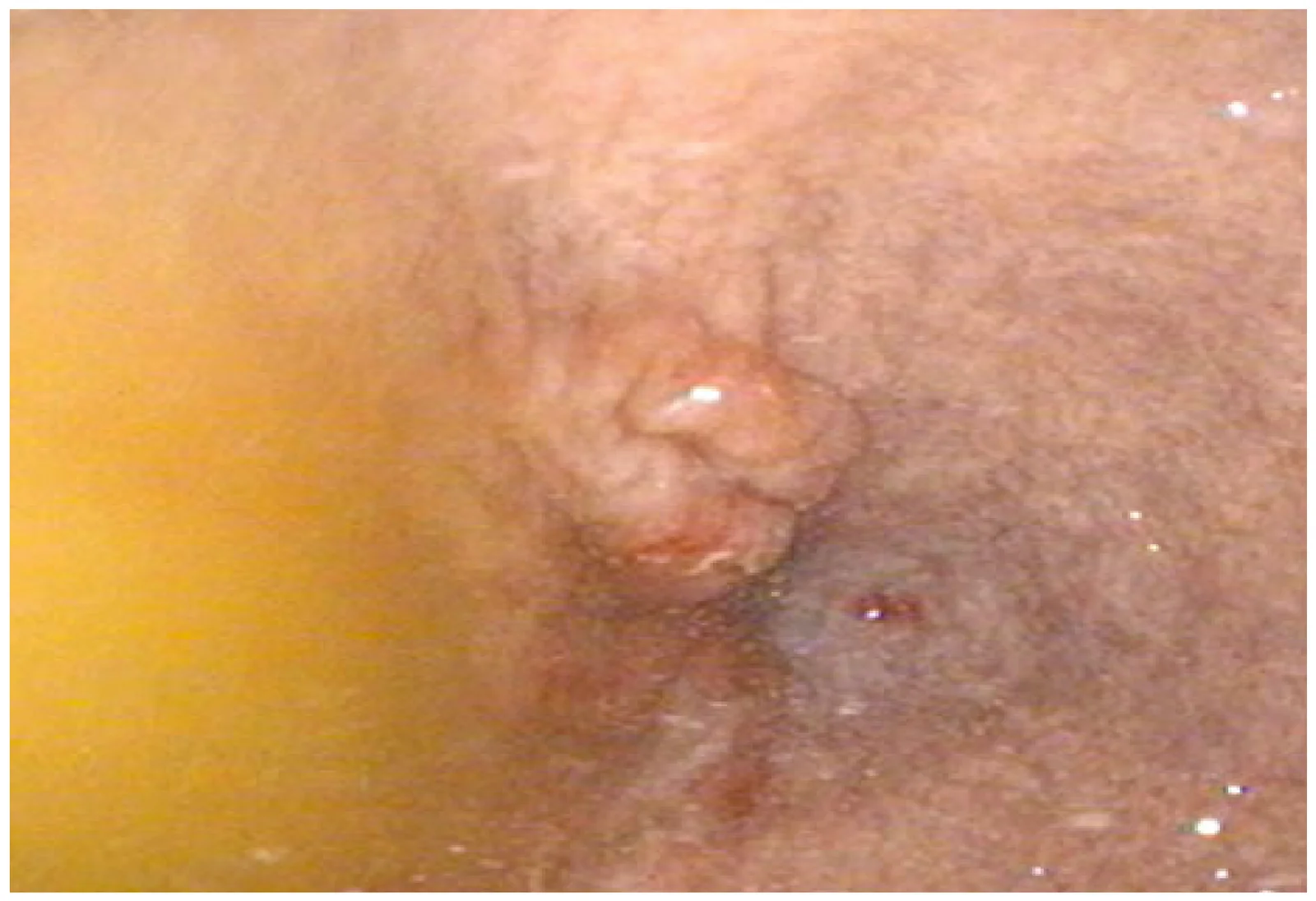
Endoscopic image of the bladder in Case One showing altered mucosa in the apex area.

An 11-year-old jumping Polish Warmblood gelding was referred to the clinic for a persistent problem characterized by stopping while ridden and assuming a urination position over the past few weeks. The owner also noted recent weight loss, despite the horse having unlimited access to roughage. A comprehensive clinical examination was conducted, revealing no pathological notes, and the heart rate, respiratory rate, and temperature were all within normal limits. The horse was sedated using acepromazine 0.05 mg/kg i.v., detomidine 0.012 mg/kg i.v., and butorphanol 0.012 mg/kg i.v. to facilitate endoscopic examinations of both the stomach and bladder. Gastroscopic examination revealed multiple grade 3/4 lesions (according to a four-stage scale of lesion severity) in the nonglandular portion, along with small, moderate, flat lesions in the pylorus. The following antiulcer treatment was initiated: omeprazole (4 mg/kg) every 24 h and misoprostol (5 mcg/kg) every 12 h. A low-sugar, low-starch diet with constant access to roughage was prescribed. During cystoscopy, a localized area of altered mucosa was observed at the bladder apex (apex vesicae). The size of the lesion was impossible to assess from the endoscopic image; the altered mucosa appeared swollen and raised (Figure 1). Samples from these lesions were collected for histopathological examination. The initial histopathological examination of the bladder biopsy sample revealed no signs or evidence of inflammation, neoplasm, or hematuria; however, scattered lymphocytes, macrophages, single mast cells, and neutrophils were noted. The horse received treatment with Phytolysin^®^, which possesses anti-inflammatory and diuretic properties and is utilized in human medicine.

Two months following the initiation of antiulcer treatment, a second gastroscopy was performed. This examination revealed significant improvement, with only a few small lesions observed in the squamous region of the stomach and mild, small areas of redness remaining in the pylorus. Despite the antiulcer treatment, the owner reported only partial improvement in the horse’s clinical signs. The undesirable behavior persisted, with the gelding still stopping during work, though it was somewhat easier to encourage him to move forward. Seven months later, the clinical signs gradually worsened. A control gastroscopy was deemed necessary and revealed new lesions in the squamous mucosa, although they were less severe than initially observed. Omeprazole therapy was reinstituted. While a reduction in the severity of clinical signs was noted, it was not as pronounced as during the first treatment course. Due to the horse’s persistent reluctance to move, a follow-up cystoscopy was performed one month later. This procedure identified a negligible amount of sand at the base of the bladder shaft (corpus vesicae). Critically, the inflammatory lesion at the top of the bladder had increased in size, and the mucosa appeared even more macroscopically altered. New samples were collected for histopathological examination. This time, the examined sections revealed inflammation with visible cell degeneration. While only a small number of epithelial cells were visible in the biopsy specimens, all the samples exhibited features consistent with chronic inflammation. No microorganisms were identified in the specimens. It is important to note that the endoscopically obtained fragments were small, and biopsies of the equine urinary bladder are relatively uncommon. Nevertheless, based on comparison with conditions observed in other mammalian species (cats, dogs, and humans), the clinical picture was deemed similar to interstitial cystitis, a condition often associated with stress and characterized by impaired mucosal healing, leading to chronic inflammation.

Dietary modifications were subsequently advised for the horse, including calcium restriction, a small addition of wheat bran to the feed, and a vitamin C supplement (2 g per os twice daily) to acidify the urine, with the aim of counteracting future sand accumulation. Decreasing urine pH is one of the described methods to slow the recurrence of sand accumulation. According to the owner’s later account, the symptoms completely resolved only after the introduction of another antiulcer therapy in conjunction with the cystitis dietary recommendations. Following this comprehensive treatment regimen, the horse was successfully used for low-level events for another six months. In the seventh month, the horse was sold, and its subsequent history remains unknown.

### 3.2. Case Two

**Figure 2:**
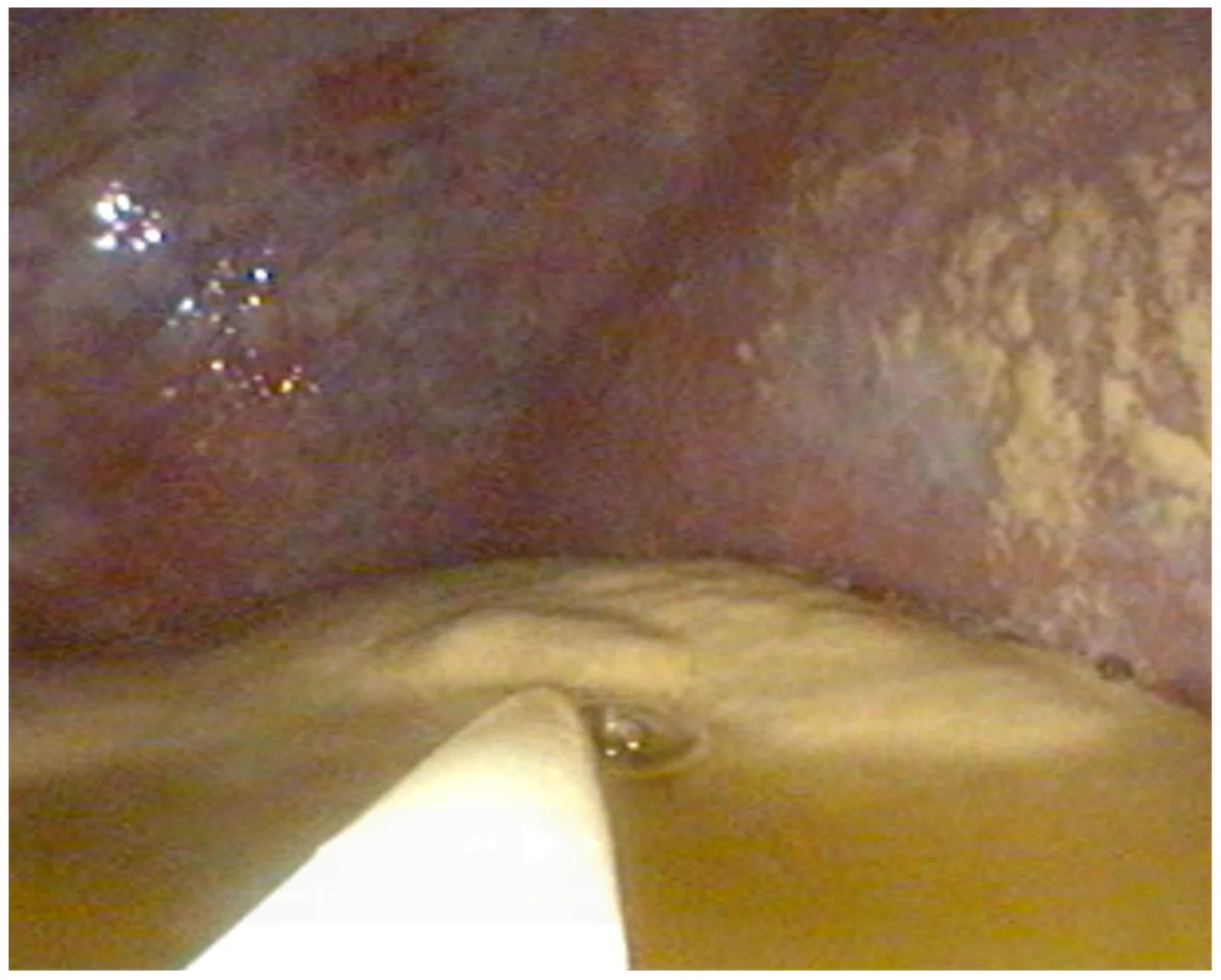
Endoscopic image of the bladder in Case Two showing sand accumulation.

A three-year-old Polish Warmblood mare was presented with concerning urinary issues, specifically involuntary urination. Upon cystoscopic examination, performed under sedation (detomidine 0.012 mg/kg i.v., butorphanol 0.012 mg/kg i.v.), the bladder lining showed sand and hemorrhagic lesions (Figure 2). Macroscopically, the urine was cloudy and blood-red. A bacteriological examination of the urine revealed Gram-positive cocci that could only be identified by sequencing. The 16S rRNA gene fragment was identified by comparison with the NCBI database, and the highest-scoring BLASTN alignment was obtained with *Ruoffia tabacinasalis* (formerly *Facklamia tabacinasalis*; GenBank accession no. OR142797.1), showing 98.71% sequence identity and 100% query coverage.

The pathogenicity of these bacteria has not yet been described. Histopathological examination revealed significant infiltration by inflammatory cells, fibrin, and numerous bacterial cells. An irregular structure was noted in the examined sections. Some cells could not be identified due to degeneration, but the tissue samples were notable for severe, patchy, irregular hyperplasia. With the degree of inflammation, a severe reaction within the epithelium would be expected. Anti-inflammatory treatment (meloxicam 0.6 mg/kg per os every 12 h) and antibiotic therapy with trimethoprim and sulfamerazine (30 mg/kg *per os* every 12 h) were administered. After the mare left the clinic, bladder lavage with 0.9% NaCl was performed several times. Six weeks later, the bacteriological examination of the urine was repeated; no pathological microorganisms were detected in the culture, but sand in the bladder was again noted. For the next few months, bladder lavages were repeated regularly. For the third time, cystoscopy was performed three months later, but there was no improvement either in endoscopy or clinical signs. The mare still had urinary incontinence, and the owner decided on euthanasia. The necropsy was not performed since the owner had not decided to do so.

### 3.3. Case Three

**Figure 3:**
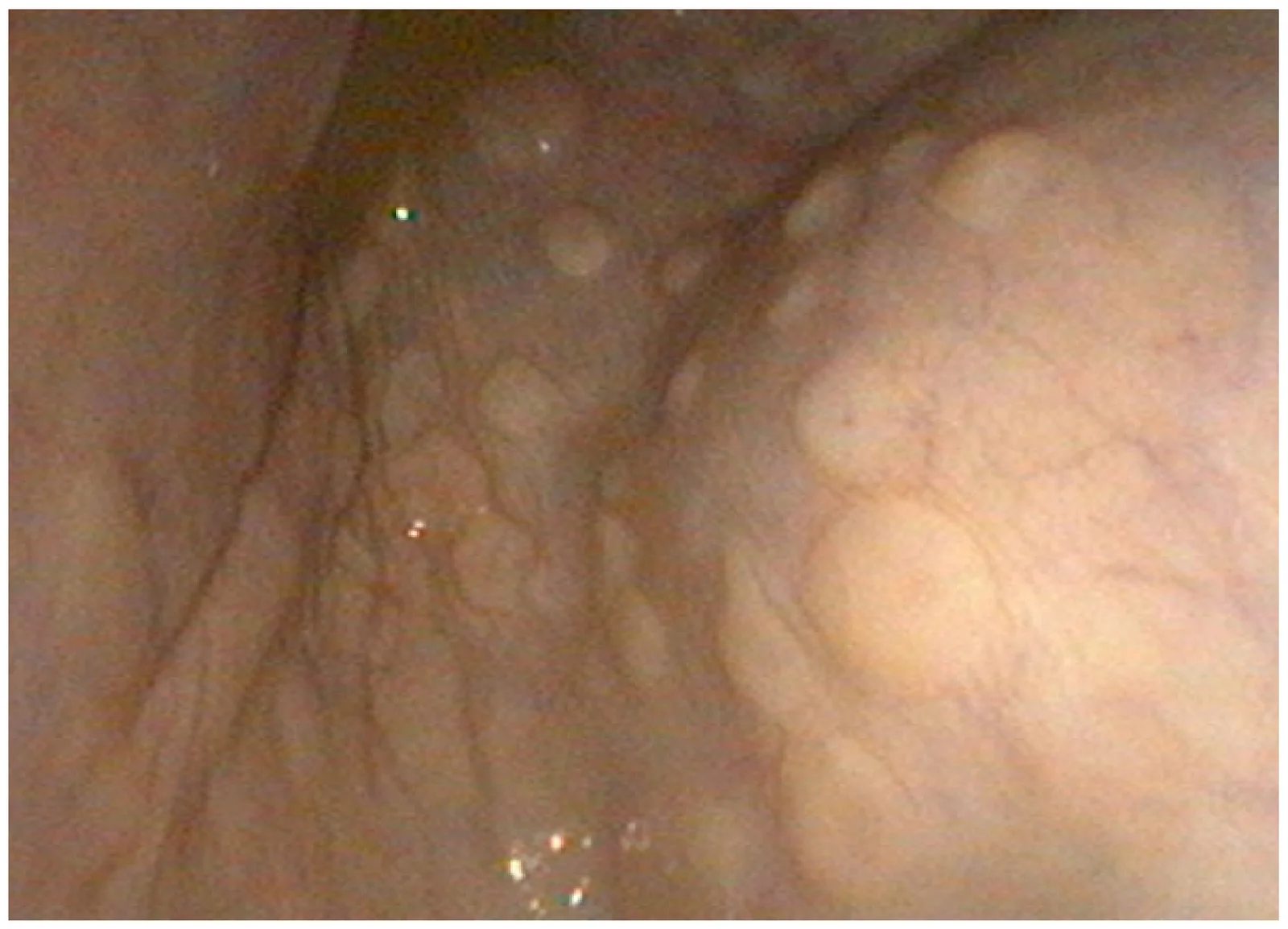
Endoscopic image of the bladder mucosa from Case Three: multifocal, round, raised lesions are visible.

A 19-year-old Polish Warmblood gelding was presented for a cystoscopic examination due to noticeable lethargy and increased urinary frequency, particularly during ridden exercise. This leisure horse, ridden by its sole owner, had a history of Equine Gastric Ulcer Syndrome (EGUS) that had previously resolved without recurrence. The gelding was maintained on a specialized diet low in sugar and starch, with *ad libitum* access to roughage, supplemented by alfalfa chaff and corn oil. The bladder endoscopy, performed under sedation (acepromazine 0.05 mg/kg i.v., detomidine 0.012 mg/kg i.v., and butorphanol 0.012 mg/kg i.v.), revealed a small amount of sand on the ventral part of the bladder, and multifocal raised lesions were observed (Figure 3). All the lesions were circular and raised, with smooth surfaces. Macroscopically, the urine had a physiological color and consistency; a full urinalysis could not be performed during the field visit. Tissue samples were taken from the lesions for histopathological examination. Lymphocytes, plasma cells, and a few neutrophils were found in the biopsy specimens, with no features of neoplasia. On the basis of the histopathological examination, a diagnosis of benign chronic cystitis with ulceration and mucosal hyperplasia was made. However, it was impossible to determine the etiology of the inflammation from this evidence alone. Therefore, no definitive diagnosis could be established because a necropsy was not performed, and the available histopathological findings were inconclusive.

A blood sample was sent to two laboratories for food allergy testing using the PAX and IgE/IgG methods, respectively. It is important to acknowledge the potential for false positive results from such tests. To mitigate the influence of inappropriate feeding on discomfort and clinical manifestations, allergy assessments were conducted prior to any dietary modifications. After that, the patient’s diet was modified. Allergens (oats, corn, soy) were excluded from the diet. Alfalfa chaff was replaced with grass chaff, and the addition of 150 g of wheat bran was recommended. The horse was treated with the herbal preparation Phytolysin^®^. This treatment was administered for two weeks at a dose of 100 g per day. Clinical improvement was noted over the following weeks, with no recurrence of clinical signs.

### 3.4. Case Four

**Figure 4:**
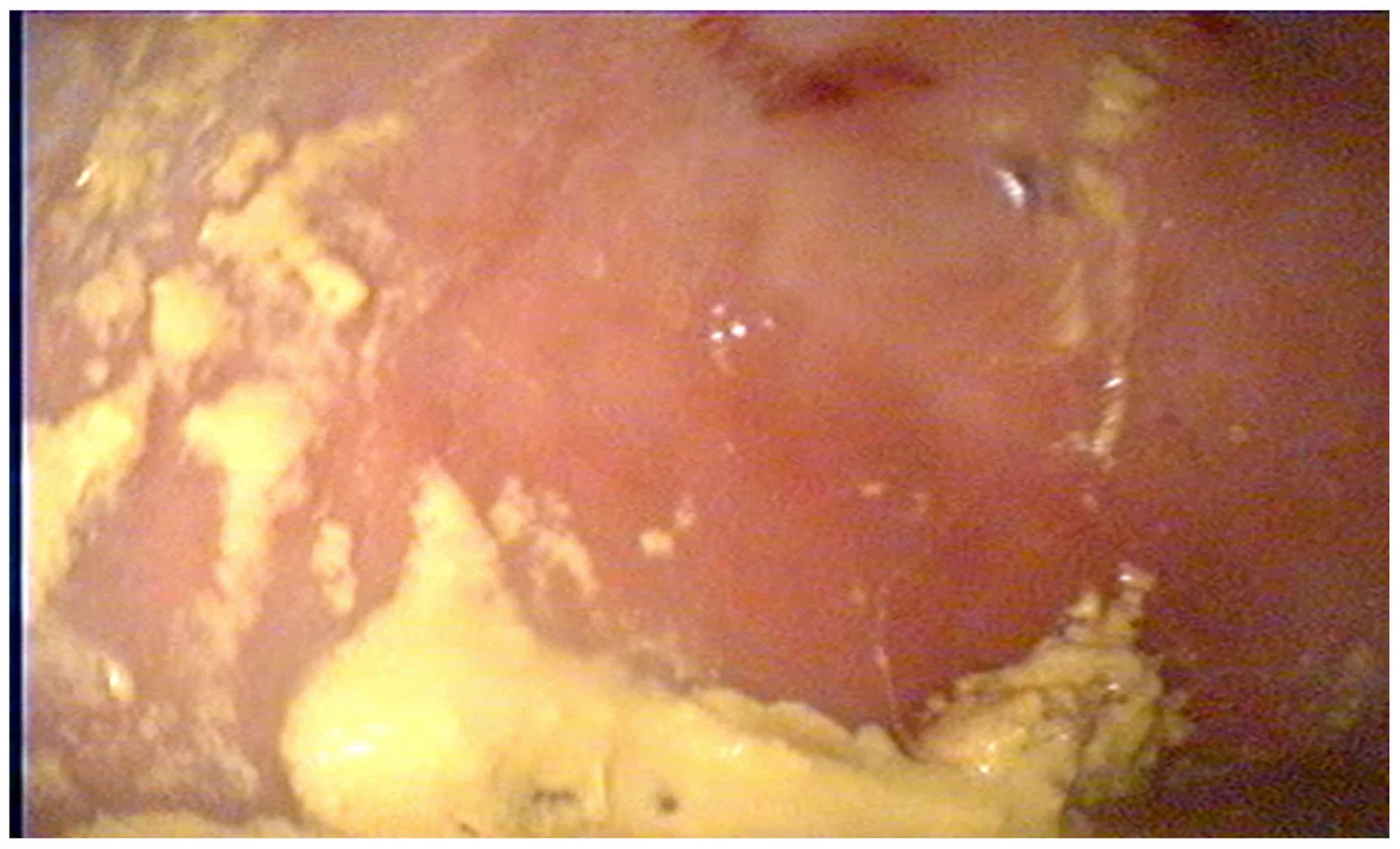
Endoscopic image of the bladder mucosa from Case Four.

A nine-year-old mare was presented to the clinic for difficulties encountered during jump training, specifically stopping in front of an obstacle and spreading her legs. A gastroscopic examination was performed under sedation (detomidine 0.012 mg/kg i.v. and butorphanol 0.012 mg/kg i.v.), which revealed small erosions on the margo plicatus and flat redness in the pylorus (Figure 4). During the same sedation, a cystoscopy was performed, revealing a small amount of sand on the bladder floor and focal mucositis, from which material was obtained for histopathological examination. The sections revealed lesions similar to those indicative of interstitial cystitis in cats. The mare was prescribed a fourteen-day course of Phytolysin^®^. Blood samples were collected, and food allergy tests were performed by two independent laboratories (using the Next+ method, which measures IgE antibodies; IgE and IgG were measured in a second laboratory). The results confirmed allergies to oats, corn, and alfalfa, which were excluded from her diet. Owing to gastric ulcer disease of the non-glandular part and mild flat redness in the pylorus, treatment with omeprazole (4 mg/kg bw. every 24 h) and sucralfate (22 mg/kg bw. every 8 h) was administered.

## 4. Discussion

The present case series expands the currently limited body of knowledge on equine lower urinary tract disorders by documenting four distinct and atypical presentations of cystitis: chronic inflammatory lesions of unknown origin, infection associated with *Facklamia* spp., and mucosal hyperplasia with ulceration. These findings highlight that cystitis in horses may be considerably more heterogeneous than traditionally recognized, and that clinically relevant bladder pathology may be overlooked when diagnostic investigations are limited to routine examinations.

From the perspective of equine internal medicine, the nonspecific nature of the presenting clinical signs, including poor performance, reluctance to move forward, urinary incontinence, and increased frequency of urination, emphasizes the importance of considering lower urinary tract disease as a differential diagnosis in horses with otherwise unexplained clinical complaints. Collectively, the integration of clinical assessment, endoscopy, histopathology, microbiological diagnostics, dietary evaluation, and longitudinal follow-up underscores the value of a precision-medicine approach to the diagnosis and management of chronic equine bladder disorders.

Herein, four cases of cystitis in horses are described. For each equine, it was impossible to determine the cause of the abnormalities found. The findings underscore that bladder anomalies, as visualized endoscopically in horses, can exhibit considerable morphological variability. One limitation of the present study was that tissue samples were obtained with endoscopic biopsy forceps. Although this approach substantially limits the size of the specimens collected, it is the only minimally invasive method for obtaining urinary bladder tissue samples. Biopsies were collected from macroscopically abnormal areas, with a minimum of three specimens obtained from each lesion to ensure adequate sampling of the mucosal layer. However, for obvious ethical and technical reasons, full-thickness bladder wall specimens could not be obtained. Cystoscopy emerges as an indispensable diagnostic tool for investigating clinical signs such as reluctance to move, urinary difficulties, or unusual posture after other possible causes, such as gastric ulcers, have been ruled out. During endoscopic examination of the bladder in horses, the most commonly diagnosed problem is submucosal cystitis, for which numerous scientific reports exist [11,12,13]. Hemorrhagic cystitis has been observed after exercise [14], and idiopathic cystitis has also been reported in horses [10]. Neoplasms, primarily squamous cell carcinoma but also transitional cell carcinoma, also occur [16].

All cases of cystitis reported in this study were confirmed by histopathological examination, and in all horses, the findings were nonspecific and previously unreported in this species. This may be related to the fact that histopathological examination of the bladder mucosa is less common in horses than in other species. In Case One, the clinical and, first and foremost, histopathological presentation is reminiscent of interstitial cystitis in cats. In cats, the syndrome is associated with a number of signs involving urinary problems, the etiology of which is multifactorial, with stress considered the main cause [17]. The condition affects older animals, most often neutered males. In addition to castration, risk factors include obesity, reduced physical activity, dry food consumption, and reduced water intake [17,20]. The causes of the signs in horse No. 1 were multifactorial, making it impossible to determine whether the discomfort associated with gastric ulcers aggravated the urinary problems or vice versa. The therapeutic approach was multimodal, making it impossible to determine which individual intervention contributed most to the observed clinical improvement. Furthermore, one inherent limitation of veterinary medicine is that patients cannot directly communicate the nature or severity of their clinical signs. Despite these limitations, the majority of horses exhibited resolution of clinical signs following treatment. Although a causal relationship cannot be definitively established, these findings suggest that cystitis was likely a contributing factor in the clinical presentation.

A urine sample from horse No. 2 contained the bacterium *Facklamia* sp. The pathogenicity of this species is poorly understood; however, there are no other reported cases in horses, and only three cases of infection in humans have been reported in the literature. The reports included a man who had overdosed on intravenous drugs and developed endocarditis. The second case involved a man who developed pharyngitis after a suicide attempt (an overdose of antidepressants). The third patient was a young woman with diabetes, chronic kidney disease, and nonalcoholic cirrhosis who was admitted with ascites and abdominal pain [21]. *Facklamia* sp. was first described in 1997 and was isolated from vaginal swabs, the endocardium, cerebrospinal fluid, bone, skin, and gallbladder [19]. The virulence of the pathogen has not yet been determined, and it often coexists with other bacteria and is isolated from patients with multiple comorbidities. When traditional laboratory methods are used, *Facklamia* can be mistaken for *Enterococcus faecalis*, *Cardiobacterium valvarum*, or *Streptococcus mutans*, so final identification is performed by deoxyribonucleic acid sequence analysis [21]. In Case Two, the pathogen was not identified in the first stage, and its presence could only be determined after sequencing. In cases three and four, lesions similar to those indicative of interstitial cystitis were confirmed by a European-certified pathology specialist. This condition, which has been described in cats, manifests as chronic urinary problems and is characterized by a distinct bladder appearance on cystoscopy, including clearly visible vascularization of the mucosa and submucosal petechiae [22,23]. Phytolysin, used as an adjunctive treatment in Cases 1 and 3, has been the subject of several studies reporting beneficial effects of Phytolysin in women with cystitis. Phytolysin was administered as an adjunct to antibiotic treatment. Although the positive effects of Phytolysin are well known in other species [24,25], in our four equine cases, it cannot be clearly concluded to what extent this compound had a positive impact, as all horses received multimodal treatment, making it impossible to distinguish the contribution of individual interventions. Therefore, further controlled studies are warranted to confirm its efficacy and safety in equine patients.

## 5. Conclusions

This case series highlights the clinical, endoscopic, microbiological, and histopathological heterogeneity of cystitis in horses and expands the currently limited knowledge of lower urinary tract diseases in horses. The four cases described herein demonstrate that chronic cystitis may present with nonspecific signs, such as poor performance, reluctance to move forward, urinary incontinence, or increased urinary frequency, potentially leading to underdiagnosis in routine equine practice. Importantly, the identification of distinct pathological phenotypes—including *Facklamia*-associated cystitis, chronic-active cystitis with mucosal hyperplasia and ulceration, and lesions closely resembling interstitial cystitis described in 19 dogs and cats—suggests that the spectrum of equine cystitis is broader than previously recognized. The clinical presentation may differ among species, but based on the histopathological image, the similarity can be confirmed, opening the way for further research in this direction. The integration of cystoscopy, histopathology, microbiological testing, dietary assessment, and longitudinal follow-up proved valuable for achieving a definitive diagnosis and guiding individualized therapeutic strategies, supporting the growing role of precision diagnostics in equine internal medicine. However, these conclusions should be interpreted with appropriate caution as they are based on only four clinical cases.

## 6. Limitations

Several limitations should be acknowledged. The study is based on only four cases, precluding conclusions regarding prevalence, risk factors, or treatment efficacy. Histopathological interpretation was constrained by the small size and superficial nature of endoscopically obtained bladder biopsy specimens, which may not fully represent larger pathological changes. Furthermore, complete follow-up data were not available for all horses, and post-mortem examination was not performed on the euthanized mares, limiting definitive characterization of the disease process. Despite these limitations, the unusual findings reported in this series provide clinically relevant insights and generate important hypotheses for future studies investigating the pathogenesis, diagnosis, and management of chronic and atypical cystitis in horses. Our case reports should be considered hypothesis-generating and warrant further investigation.

## Data Availability

All data are mentioned in the main article.

## Abbreviation

The following abbreviation is used in this manuscript:

FUS	Feline urologic syndrome
